# Supplementary material for: Specific Amino Acid Substitutions in OXA-51-Type β-Lactamase Enhance Catalytic Activity to a Level Comparable to Carbapenemase OXA-23 and OXA-24/40
Source: Int J Mol Sci. 2022 Apr 19;23(9):4496. doi: 10.3390/ijms23094496 (PMC9105447; doi:10.3390/ijms23094496)
Supplement: Supplementary file 1 [file ijms-23-04496-s001.zip › ijms-1695780-supplementary.pdf]

Article

Specific Amino Acid Substitutions in OXA-51-Type *β*-Lactamase Enhance Catalytic Activity to a Level Comparable to Carbapenemase OXA-23 and OXA-24/40

Kwan-Wai Chan [1,2,†], Chen-Yu Liu [2,†], Ho-Yin Wong [1], Wai-Chi Chan [1], Kwok-Yin Wong [1] and Sheng Chen [2,*]

[1] State Key Laboratory of Chemical Biology and Drug Discovery, The Hong Kong Polytechnic University, Hung Hom, Kowloon, Hong Kong; bill.kw.chan@connect.polyu.hk (K.-W.C.); marcus-ho-yin.wong@polyu.edu.hk (H.-Y.W.); edwardwchan@yahoo.com.hk (W.-C.C.); kwok-yin.wong@polyu.edu.hk (K.-Y.W.)

[2] Department of Infectious Diseases and Public Health, City University of Hong Kong, Kowloon, Hong Kong; chenyliu4-c@my.cityu.edu.hk

* Correspondence: shechen@cityu.edu.hk; Tel.: +852-3442-5782

† These authors contributed equally to this work.

Keywords: *A. baumannii*; carbapenem resistance; carbapenem-hydrolyzing class D β-lactamases (CHDLs); OXA-51 variants; structure/function relationship

Table S1. Characteristics of 14 *A. baumannii* clinical strains included in this study.

Strain	Year and Place of Isolation	ST Type (Oxford)	International Clone	Presence of Resistance Determinants						
				carO	*adeB*	*adeRS*	*bla*$_{OXA-51}$ Variants	IS*Aba1*-*bla*$_{OXA-51}$	*bla*$_{OXA-24/40}$	IS*Aba1*-*bla*$_{OXA23}$
AB1	2004, Hong Kong	208	IC II	+	+	-	OXA-83	+	-	-
AB2	2008, Hong Kong	940	Non-IC II	+	+	+	OXA-99	-	OXA-23	+
AB3	2011, Hong Kong	208	IC II	+	-	-	OXA-66	+	OXA-23$^{\#}$	+
AB4	2011, Hong Kong	253	IC II	+	+	+	OXA-66	-	OXA-23	+
AB5	2004, Hong Kong	940	Non-IC II	+	+	+	OXA-99	-	OXA-23	+
AB7	2011, Hong Kong	208	IC II	+	+	+	OXA-82	+	-	-
AB8	2003, Hong Kong	208	IC II	+	+	+	OXA-82	+	-	-
AB10	2000, Hong Kong	208	IC II	+	+	+	OXA-66	-	-	-
MH1	2000, Hong Kong	684	IC II	+	+	+	OXA-66	-	-	-
MH2	2010, Hong Kong	218	IC II	+	+	+	OXA-66	-	OXA-72	-
MH3	2011, Hong Kong	208	IC II	+	+	+	OXA-79	+	-	-
MH5	2006, Hong Kong	940	Non-IC II	+	-	-	OXA-99	-	OXA-23	+
MH6	2013, Henan, China	195	IC II	+	+	+	OXA-66	-	OXA-23	+
MH7	2013, Henan, China	369	IC II	+	+	+	OXA-66	-	OXA-23$^{\#}$	+

Presence of endogenous genes and other resistance determinants were obtained from analysis of whole genome sequences. $^{\#}$ Chromosomal located.
